# NEIL3 Mediates Lung Cancer Progression and Modulates PI3K/AKT/mTOR Signaling: A Potential Therapeutic Target

**DOI:** 10.1155/2022/8348499

**Published:** 2022-04-30

**Authors:** Hongbo Huang, Qingwang Hua

**Affiliations:** ^1^Department of Thoracic Surgery, Hwa Mei Hospital, University of Chinese Academy of Sciences, Ningbo, Zhejiang, China; ^2^Ningbo Institute of Life and Health Industry, University of Chinese Academy of Sciences, Ningbo, Zhejiang, China

## Abstract

**Background:**

Nei endonuclease VIII-like 3 (NEIL3) is widely involved in pathophysiological processes of the body; however, its role in lung cancer has not been conclusively determined.

**Objective:**

This study is aimed at exploring the role of NEIL3 in lung cancer.

**Methods:**

The public data used in this study were downloaded from The Cancer Genome Atlas (TCGA) database. “Limma” in R was used for the analysis of differentially expressed genes. Clinical correlations and prognostic analyses were performed using the survival package in R. The proliferative abilities of lung cancer cells were evaluated by the CCK8 and colony formation assays while their invasive and migration abilities were assessed by the transwell and wound healing assays. Quantitative real-time PCR (qRT-PCR) and western blot analyses were utilized to detect RNA and protein levels. Biological differences between groups were determined by gene set enrichment analysis (GSEA). Tumor Immune Dysfunction and Exclusion (TIDE) as well as Genomics of Drug Sensitivity in Cancer (GDSC) was used for immunotherapeutic and chemotherapeutic sensitivity analyses.

**Results:**

NEIL3 was upregulated in NSCLC tissues and cell lines, implying that it is involved in lung cancer initiation and progression. Clinical correlation and prognostic analyses showed that NEIL3 was associated with worse clinical features (stage and T and N classifications) and poor prognostic outcomes. *In vitro*, NEIL3 significantly enhanced NSCLC proliferation, invasion, and migration. GSEA indicated that NEIL3 might be involved in PI3K/AKT/mTOR, G2/M checkpoints, and E2F target pathways. Inhibition of NEIL3 suppressed cyclinD1 and p-AKT protein levels; however, it had no effects on AKT levels, indicating that NEIL3 can partially activate the PI3K/AKT/mTOR signaling pathway. The predicted result of TIDE indicated that immunotherapeutic nonresponders had elevated NEIL3 levels. Moreover, there was a positive correlation between NEIL3 levels and chemosensitivity to cisplatin and paclitaxel.

**Conclusion:**

In general, NEIL3 mediates NSCLC progression and affects sensitivity to immunotherapy and chemotherapy; therefore, it is a potential molecular target for treatment.

## 1. Introduction

Globally, lung cancer is the leading cause of cancer-associated mortality, accounting for approximately 2.2 million new cases and 1.8 million deaths in 2020 [1]. The most frequent pathological lung cancer subtype is non-small-cell lung cancer (NSCLC), which consists of lung squamous cell carcinoma (LUSC) and lung adenocarcinoma (LUAD) [2]. As a multistep and multifactorial disease, lung cancer is associated with both environmental and genetic factors, including smoking, lifestyle, or underlying disease [3]. Although surgical resection combined with chemoradiotherapy can effectively improve the prognostic outcomes for early-stage lung cancer patients, their efficacies in advanced and metastatic lung cancer patients are limited [4]. Immunotherapy is becoming increasingly important in advanced solid tumors [5]. In lung cancer, immune checkpoint inhibitors have shown promising therapeutic outcomes [6, 7]. Currently, the treatment paradigm for lung cancer has shifted to targeted therapy [8]. Emerging biomarkers have been incorporated into the management of NSCLC patients. For instance, a comprehensive review conducted by Rodríguez et al. indicated that a combination of effective biomarkers with risk stratification, clinical data, radiomics, molecular information, and artificial intelligence has the potential to improve clinical decision-making [9]. Some specific biomarkers, including EGFR, ALK, and ROS mutations, have a high clinical significance [10]. Ostrin et al. documented that biomarker tests such as Early CDT-Lung, Nodify XL2, and Percepta have a great potential in improving the early detection of NSCLC [11]. Therefore, identification of effective molecular targets for lung cancer therapy is a necessity.

Nei endonuclease VIII-like 3 (NEIL3), a class of DNA glycosylases homologous to the bacterial Fpg/Nei family, are involved in diverse physiological and pathophysiological processes [12]. For instance, Karlsen et al. found that in mouse models, NEIL3 deletion remodeled the intestinal microbial flora and increased intestinal permeability. These metabolic alterations play a role in atherogenesis [13]. At the cellular level, Zhou et al. revealed that NEIL3 repairs telomere damage during the S/G2 phase to secure chromosomal segregation at mitosis, protecting genomic stability [14]. In addition, NEIL3 is involved in human malignancy. Wang et al. reported that NEIL3 contributes towards liver cancer carcinogenesis by regulating the PI3K/Akt/mTOR signaling [15]. Moreover, Tran et al. found that NEIL3 might be associated with genomic mutations and chromosomal variations [16]. Tumors with overexpressed NEIL3 are often accompanied by anomalous expression levels of homologous recombination genes (BRCA1/2) and mismatch repair genes (MSH2/MSH6) [16]. Wang et al. found that NEIL3 promotes prostate cancer cell proliferation and cisplatin resistance by inhibiting the phosphorylation of ATR and ATM serine/threonine kinase [17]. Meanwhile, Klattenhoff et al. found that the loss of NEIL3 markedly enhances replication-associated double-strand breaks and enhances sensitivity to ATR inhibitors in glioblastoma cells [18]. However, the role of NEIL3 in NSCLC has not been conclusively determined.

Data generated by next-generation sequencing along with bioinformatics development provides great convenience for molecular target identification. Through a series of bioinformatics analyses, we identified NEIL3 as a gene of interest. We also found that NEIL3 was overexpressed in NSCLC tissues as well as cell lines and that it was correlated with worse clinical features and poor prognostic outcomes for NSCLC patients. *In vitro*, NEIL3 significantly promoted NSCLC cell proliferation, invasion, and migration by regulating the PI3K/AKT/mTOR signaling pathway. Moreover, NEIL3 affects immunotherapeutic and chemotherapeutic sensitivity, making it a potential therapeutic target for NSCLC patients.

## 2. Methods and Materials

### 2.1. Acquisition and Analysis of Open-Access Data

The open-access gene expression data for NSCLC patients were downloaded from The Cancer Genome Atlas (TCGA) database (2022-1-18, https://portal.gdc.cancer.gov/, TCGA-LUAD, TCGA-LUSC). Gene annotation was completed based on the genomic reference file downloaded from the ENSEMBL website (http://asia.ensembl.org/index.html). The expression matrix was in the form of FPKM, which was converted into the TPM form based on R code. Data were preprocessed and normalized before analysis. Clinical information for TCGA patients, including patient ID, survival time, survival status, gender, age, clinical stage, and TNM classifications, was extracted using R code. “Limma” in R was used for differential expressed gene (DEG) analyses in two groups with the threshold of |logFC > 1| and adjusted *p* < 0.05. Survival package was used to perform survival analysis of NEIL3. The inclusion criteria were (i) samples from patients with clinically diagnosed NSCLC (LUAD and LUSC), (ii) samples with complete clinical information and transcriptional profiling, and (iii) open-accessed samples. The exclusion criteria were (i) patients whose pathology was not NSCLC, (ii) samples without complete clinical information and/or transcriptional profiling, and (iii) samples that could not be open-accessed. Only the samples that met our criteria were included in our analysis.

### 2.2. Protein-Protein Interaction Network

To explore the underlying interactions, the symbol of the selected gene was uploaded to the STRING website (https://cn.string-db.org/cgi/input?sessionId=WjOlJ4PUsdS5&input_page_show_search=on). Cytoscape v3.7.2 was used for network visualization. Based on the number of connections of a node, the top 20 significant nodes were identified using the cytoHubba plug-in.

### 2.3. Gene Set Enrichment Analysis (GSEA)

GSEA, which was completed using fgsea and clusterProfiler package in R, was performed to evaluate biological differences between low and high NEIL3-expressing NSCLC patients. The reference pathway list was Hallmark.gmt.

### 2.4. Immune Infiltrations and Response Analysis

Immune infiltration analyses were performed using the CIBERSORT deconvolution algorithm (available online: https://cibersort.stanford.edu/), which could quantify the abundance of 22 specific cell types. Based on gene expression patterns, Tumor Immune Dysfunction and Exclusion (TIDE) analysis was used to assess immunotherapeutic responses among NSCLC patients.

### 2.5. Drug Sensitivity Analysis

Correlations between NEIL3 and IC50 (half maximal inhibitory concentrations) for several chemotherapeutic drugs were evaluated based on the Genomics of Drug Sensitivity in Cancer (GDSC) database. This tool predicts treatment responses for each patient based on sample transcriptome.

### 2.6. Cell Lines, Tissue, and Quantitative Real-Time PCR (qRT-PCR)

Human bronchial epithelial cell lines (BEAS-2B) and lung cancer cell lines (SPC-A-1, SK-MES-1, A549, and H1299) were acquired from routinely cultured laboratory stocks. The SPC-A-1 cell line was cultured in DMEM with 10% FBS while the other cell lines were cultured in 1640 medium with 10% FBS. Cells were grown under standard cell culture conditions (37°C, 5% CO_2_). Four paired lung cancer and adjacent tissues were collected from HwaMei Hospital. All the patients signed consent forms, and the protocol was approved by the Research Ethics Committee of HwaMei Hospital, according to the Helsinki Declaration. Total RNA was extracted using the TRIzol reagent and reverse transcribed into cDNA using a Reverse Transcription Kit (K1691, ThermoFisher Scientific). The SyBr Green PCR system was used to perform qRT-PCR. The primers used in this study were the following: NEIL3, forward 5′-GTCCTTCCCATTCTGCAACC-3′ and reverse 5′-GAAACAGAGGAGGACCAAACA-3′, and GAPDH, forward 5′-GCAAATTCCATGGCACCGT-3′ and reverse 5′-TCGCCCCACTTGATTTTGG-3′.

### 2.7. Cell Transfections

A Lipofectamine 2000 transfection kit (11668019, Invitrogen) was used for cell transfection, as instructed by the manufacturer. The NEIL3 shRNA and control plasmids were purchased from Genepharma (Suzhou, China). Target sequences were the following: siRNA1: 5′-GCCTGTTTAATGGATATGTTT-3′; siRNA2: 5′-CTGTTAAAGTTTGTCAATTAA-3′; and siRNA3: 5′-GACGATAAAGTGTTTTTAGTA-3′.

### 2.8. Western Blot

Extraction of total proteins was done using the total protein extraction kit (P0027, Beyotime), as instructed by the manufacturer. Primary antibodies, including AKT (1 : 5000), cyclinD1 (1 : 5000), phosphorylated AKT (1 : 2000), and GAPDH (1 : 20000) were purchased from Cell Signaling Technologies (Danvers, MA). The PVDF membrane was used to perform the western blot assay, following a standardized process.

### 2.9. Cell Proliferation Assay

The CCK8 and colony formation assays were performed to evaluate cancer cell proliferative abilities. For the colony formation assay, cells were resuspended and seeded in a six-well plate. After 14 days of incubation at 4°C, cells were fixed and stained using crystal violet. The CCK8 assay was performed using a CCK8 kit (CK04, Dojindo, Shanghai, China), as instructed by the manufacturer.

### 2.10. Transwell Assay

This assay was performed using the transwell chamber with a pore size of 8 *μ*m and 24-well plates. Briefly, the transwell chamber separated the plate into upper and lower chambers. The upper chamber was seeded with 1 × 10^4^ cells in a medium without FBS while the lower chamber was supplemented with a medium containing 20% FBS. After 24 h, cells were fixed in 4% paraformaldehyde and stained with crystal violet.

### 2.11. Wound Healing Assay

Cells were seeded in a six-well plate and incubated to 90% density. Then, scratches were made on cells in each well using a 10 *μ*l pipette tip. The original medium was removed, and the medium without FBS was added. After 24 h, wound healing was microscopically observed.

### 2.12. Statistical Analysis

Statistical analyses were completed using R and GraphPad Prism 8 software. Normally distributed variables were analyzed using the Students *T*-test while nonnormally distributed variables were analyzed using Kruskal-Wallis. *p* ≤ 0.05 was set as the threshold for statistical significance.

## 3. Results

### 3.1. Identification of the Key Gene in NSCLC

**Figure 1:**
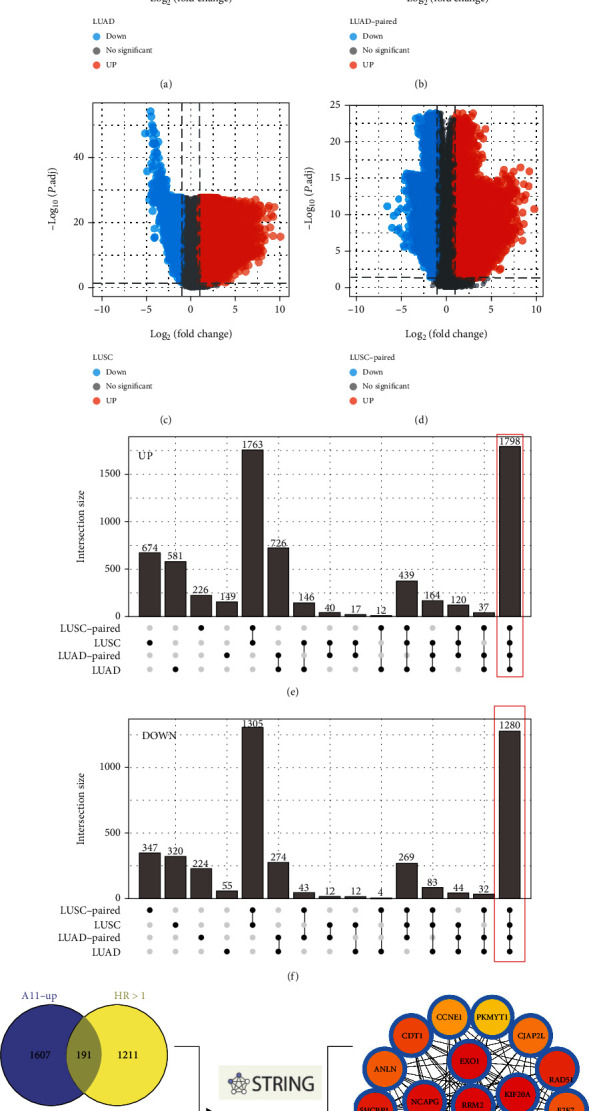
Identification of NEIL3 as the interested gene in our study. Notes: (a–d) DEGs analysis was performed between the tumor and normal tissues in TCGA-LUAD and TCGA-LUSC; (e) a total of 1798 genes were commonly upregulated in TCGA-LUAD and TCGA-LUSC, as well as in paired samples; (f) a total of 1280 genes were commonly downregulated in TCGA-LUAD and TCGA-LUSC, as well as paired samples; (g) top 20 key nodes of the commonly upregulated and downregulated genes based on the PPI network.

First, we performed differential expression analysis between TCGA nonpaired and paired tissues with the threshold of |logFC > 1| and adjusted *p* < 0.05, including TCGA-LUAD (Figures 1(a) and 1(b)) and TCGA-LUSC (Figures 1(c) and 1(d)). A total of 1798 genes were commonly upregulated while 1280 genes were commonly downregulated (Figures 1(e) and 1(f)). Furthermore, univariate cox regression analysis was performed to identify prognosis-related genes. Among the 1798 upregulated genes, 191 genes were risk factors while among the 1280 downregulated genes, 84 were protective genes (Figure 1(g)). Based on these genes, the PPI network was constructed, and the top 20 significant nodes were visualized. Among them, NEIL3 has not been fully explored in NSCLC, and therefore, it earned our interest (Figure 1(g)).

### 3.2. NEIL3 Was Upregulated in NSCLC Tissues and Was Correlated with Aggressive Clinical Features

**Figure 2:**
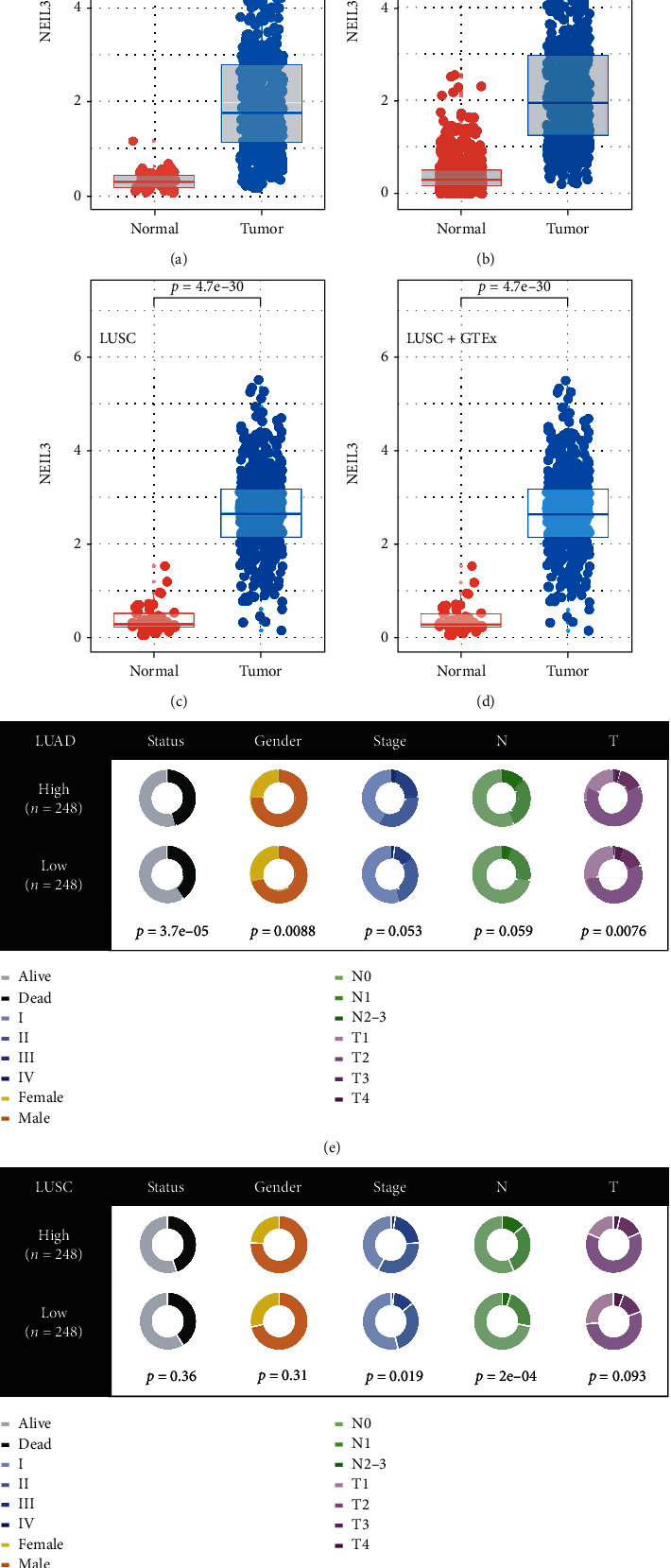
NEIL3 was upregulated in lung cancer and was associated with worse clinical features. Notes: (a–d) a higher NEIL3 expression was found in LUAD, LUAD+GTEx, LUSC, and LUSC+GTEx cohorts; (e, f) clinical correlation of NEIL3 in TCGA-LUAD and TCGA-LUSC.

**Figure 3:**
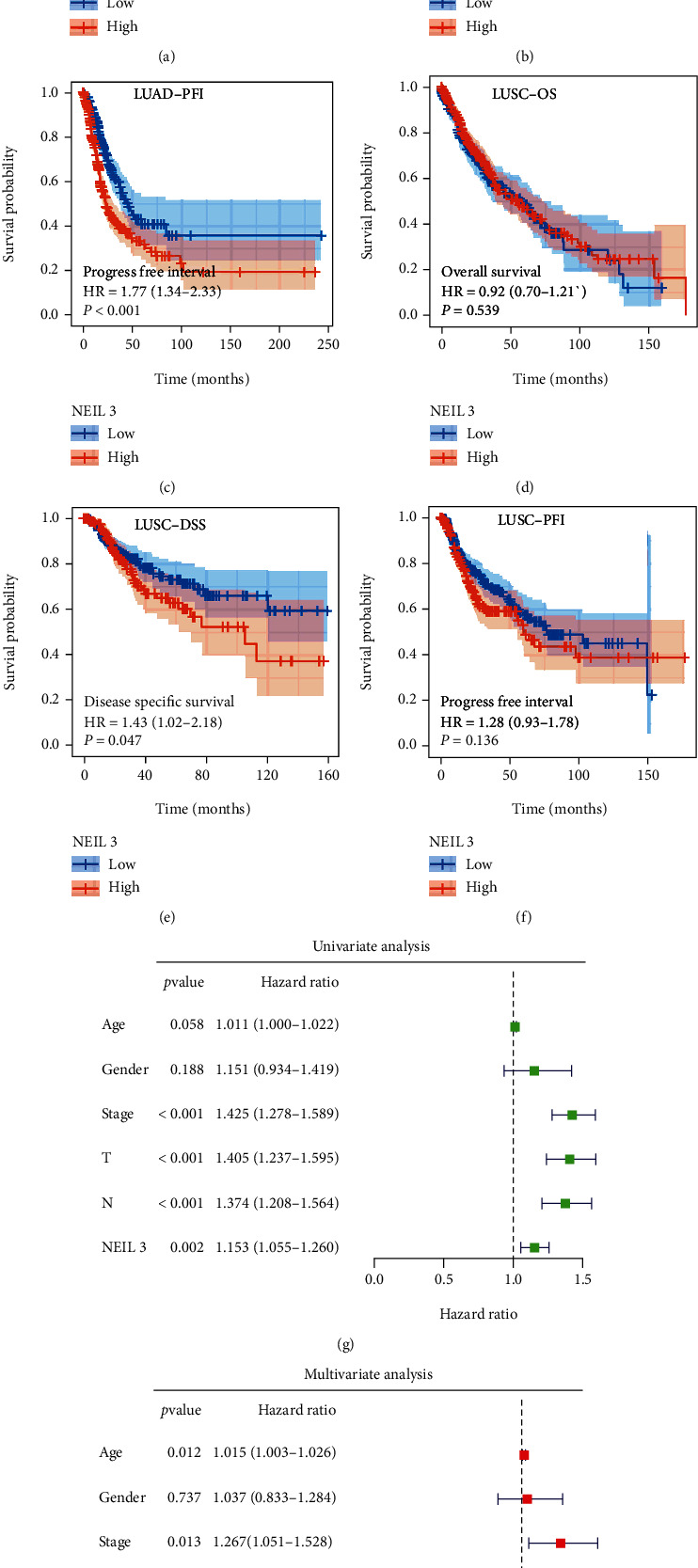
NEIL3 was associated with poor prognostic outcomes in lung cancer patients. Notes: (a–c) association between NEIL3 and patient OS, DSS, and PFI in the TCGA-LUAD cohort; (d–f) association between NEIL3 and patient OS, DSS, and PFI in the TCGA-LUSC cohort; (g, h) univariate and multivariate analyses indicated that NEIL3 was an independent prognostic marker in lung cancer patients.

Based on TCGA and GTEx data, NEIL3 was markedly upregulated in NSCLC (Figures 2(a)–2(d); TCGA-LUAD, *p* < 0.001; TCGA-LUAD+GTEx, *p* < 0.001; TCGA-LUSC, *p* < 0.001; and TCGA-LUSC, *p* < 0.001). Patients with elevated NEIL3 levels were associated with worse clinical features, including clinical stage and T and N classifications (Figures 2(e) and 2(f)). In the TCGA-LUAD cohort, patients with elevated NEIL3 levels had significantly short overall survival (OS), disease-specific survival (DSS), and progression-free interval (PFI) (Figure 3(a), OS, HR = 1.98, *p* < 0.001; Figure 3(b), DSS, HR = 2.30, *p* < 0.001; and Figure 3(c), PFI, HR = 1.77, *p* < 0.001). Differences in OS in the TCGA-LUSC cohort were not significant (Figure 3(d), OS, HR = 0.92, *p* = 0.539). However, NEIL3 markedly affected the DSS of patients in the TCGA-LUSC cohort (Figure 3(e), DSS, HR = 1.43, *p* = 0.047). There was a clear separation between PFI KM curves of high and low NEIL3-expressing patients (Figure 3(f), PFI, HR = 1.28, *p* = 0.136). Univariate and multivariate analyses showed that independent of other clinical features, NEIL3 is an effective prognostic biomarker (Figures 3(g) and 3(h)).

### 3.3. NEIL3 Promotes NSCLC Cell Proliferation, Invasion, and Migration

**Figure 4:**
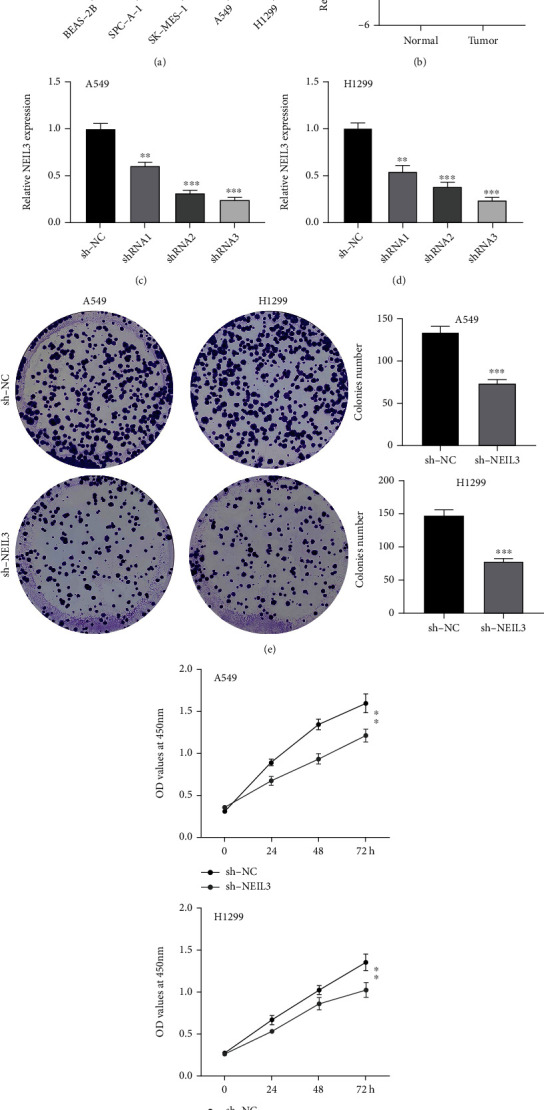
NEIL3 promoted lung cancer cell proliferation. Notes: (a) expression levels of NEIL3 in lung cancer cell lines; (b) expression levels of NEIL3 in lung cancer tissue; (c, d) qRT-PCR was performed to evaluate the knockdown efficiency of NEIL3 in cancer cell lines; (e) colony formation assay was performed in control and NEIL3 knockdown cells; (f) CCK8 assay was performed in control and NEIL3 knockdown cells.

**Figure 5:**
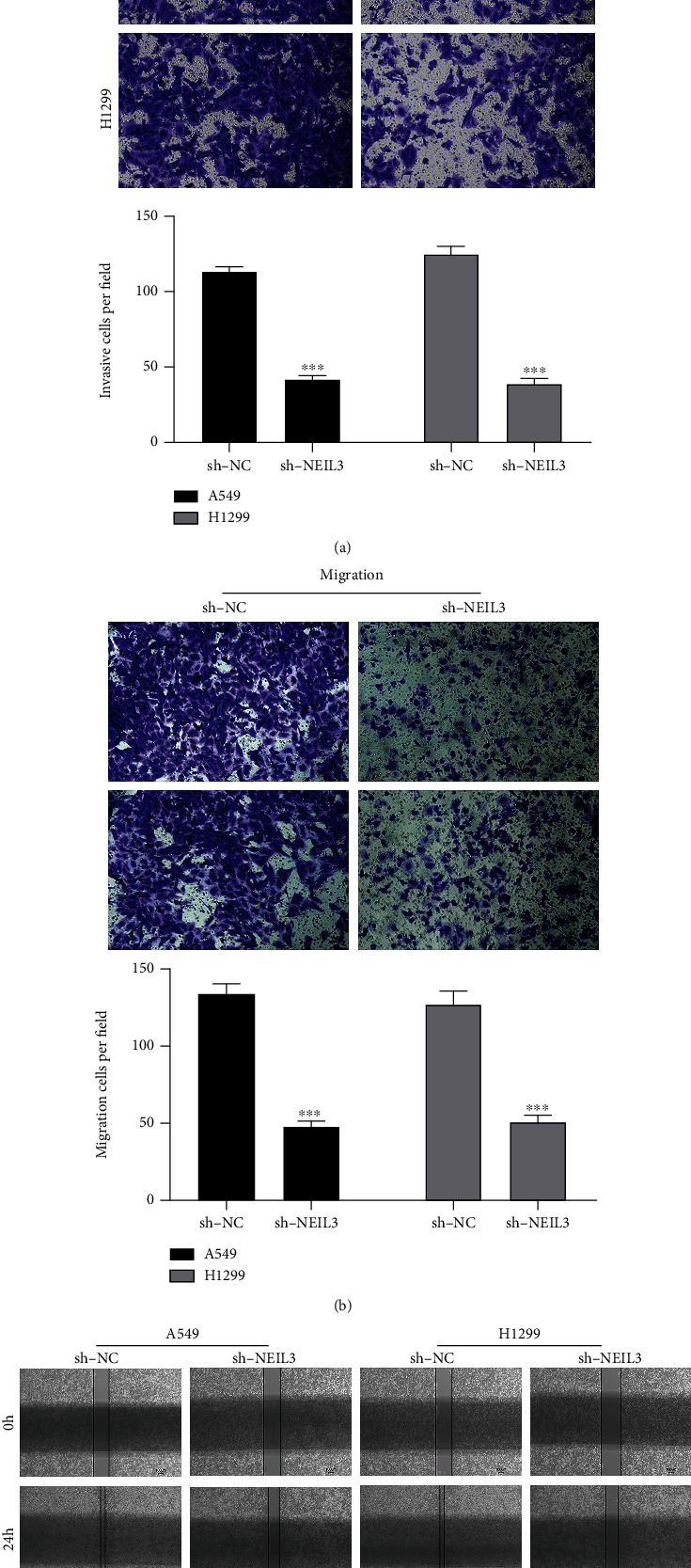
ENO2 facilitated lung cancer cell invasion and migration. Notes: (a, b) transwell assay was performed in control and NEIL3 knockdown cells; (c) wound healing assay was performed in control and NEIL3 knockdown cells.

Previous clinical correlation analysis indicated that NEIL3 was associated with worse clinical features. Therefore, we evaluated the potential role of NEIL3 at the cellular level. We found that NEIL3 was significantly upregulated in NSCLC cell lines, compared to normal cell lines (Figure 4(a)). Meanwhile, a higher NEIL3 mRNA expression level was also observed in lung cancer tissue (Figure 4(b)). Among the NSCLC cell lines in which NEIL3 was elevated, A549 and H1299 cell lines were selected for further assays. Knockdown efficiency of shRNA3 was validated by qRT-PCR (Figures 4(c) and 4(d)). Colony formation and CCK8 assays showed that NEIL3 knockdown markedly suppressed NSCLC cell proliferations (Figures 4(e) and 4(f)). The transwell and wound healing assays showed that NEIL3 knockdown markedly inhibited NSCLC cell invasion and migration abilities (Figures 5(a) and 5(c)).

### 3.4. NEIL3 Regulated the Activities of the PI3K/AKT/mTOR Signaling Pathway

**Figure 6:**
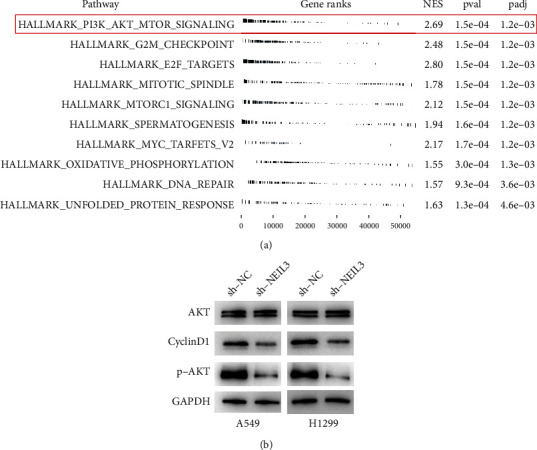
NEIL3 regulated the activation of the PI3K/AKT/mTOR signaling pathway. Notes: (a) GSEA in patients with low and high NEIL3 expressions; (b) western blot assay was performed to detect the key molecule of PI3K/AKT/mTOR signaling pathway in control and NEIL3 knockdown cells.

GSEA was performed to identify biological differences between the high and low NEIL3-expressing patients. In patients with high NEIL3 expressions, the PI3K/AKT/mTOR pathway, G2/M checkpoints, and E2F targets were aberrantly activated (Figure 6(a)). A previous study reported that NEIL3 can affect the activities of the PI3K/AKT/mTOR signaling pathway [15]. Therefore, we evaluated whether the cancer-promoting effects of NEIL3 in NSCLC are mediated by PI3K/AKT/mTOR signaling. NEIL3 knockdown significantly suppressed the protein levels of cyclinD1 and p-AKT; however, it had no effects on AKT protein levels, indicating that NEIL3 partially activates the PI3K/AKT/mTOR signaling pathway (Figure 6(b)).

### 3.5. NEIL3 Regulates Immunotherapeutic and Chemotherapeutic Sensitivity

**Figure 7:**
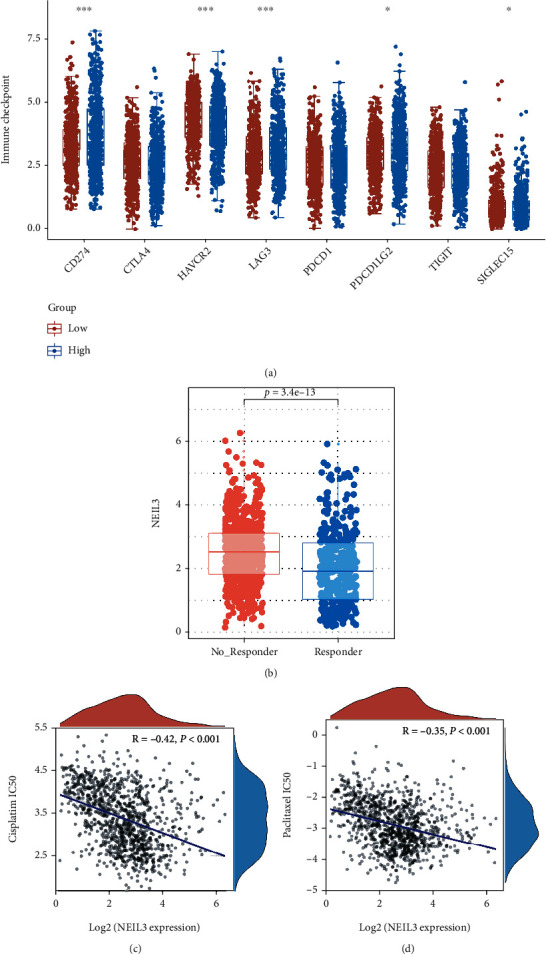
NEIL3 was associated with sensitivity to immunotherapy and chemotherapy in lung cancer patients. Notes: (a) correlation between NEIL3 and several immune checkpoint genes; (b) expression levels of NEIL3 in immunotherapeutic responsive and nonresponsive patients; (c, d) patients with higher NEIL3 might be more sensitive to cisplatin and paclitaxel.

Immunotherapy is a promising prospect in lung cancer treatment. We evaluated the correlation between NEIL3 and several key immune checkpoint molecules. Patients with elevated NEIL3 levels had higher CD274, LAG3, and PDCD1LG2 expressions as well as suppressed HAVCR2 and SIGLEC15 levels (Figure 7(a)). TIDE revealed that immunotherapeutic nonresponders had elevated NEIL3 levels, indicating that NEIL3 may inhibit the response rate of NSCLC to immunotherapy (Figure 7(b)). Cisplatin and paclitaxel are common chemotherapeutic options for lung cancer. Therefore, we performed drug sensitivity analysis through the GDSC database to identify the underlying effects of NEIL3 on chemotherapeutic sensitivity. We found that NEIL3 significantly increased sensitivity to cisplatin and paclitaxel (Figures 7(c) and 7(d); cisplatin IC50, *R* = −0.42, *p* < 0.001; paclitaxel: *R* = −0.35, *p* < 0.001).

## 4. Discussion

Globally, lung cancer has the highest morbidity and mortality rates. Therefore, it is imperative to identify novel and effective targets for lung cancer diagnosis and treatment.

NEIL3, a class of DNA glycosylases, is involved in DNA repair through DNA base excision repair [19]. Moreover, genomic instability is a crucial factor in cancer, significantly contributing to cancer progression [20]. Given the biological effects of NEIL3 on genomic stability, it may have oncogenic effects. The underlying mechanisms of NEIL3 in cancer have attracted extensive attention, making it an attractive therapeutic target for specific cancers [15]. Zhao et al. found that NEIL3 prevents senescence in liver cancer by repairing oxidative lesions at telomeres during mitosis, contributing to poor prognostic outcomes [21]. Moreover, Rolseth et al. found that compared to control mice, cancer predisposition or increased spontaneous mutation frequencies were not observed in NEIL1/2/3 DNA glycosylases-deficient mice, indicating that NEIL1/2/3 might be essential in cancer prevention [22]. The cancer-promoting effects of NEIL3 have been proven in multiple cancers, including prostate, liver, and breast cancers [17, 21, 23]. Our results indicate that NEIL3 promotes NSCLC cell proliferation, invasion, and migration partially by regulating the classic PI3K/AKT/mTOR signaling pathway; thus, it might be a promising therapeutic target.

GSEA showed that NEIL3 is involved in PI3K/AKT/mTOR, G2/M checkpoints, and E2F target signaling. The PI3K/AKT/mTOR signaling pathway plays an important role in development and tumorigenesis [24]. In lung cancer, PI3K/AKT/mTOR signaling is a critical regulatory axis contributing to cell malignant phenotypes and drug resistance [25]. The G2/M checkpoint is an essential step in the cell cycle. Hung et al. found that bavachinin induces G2/M cell cycle arrest and apoptosis of NSCLC cells through the ATM/ATR signaling pathway [26]. Moreover, Yao et al. indicated that cyclin K depletion suppresses lung cancer cell proliferation and defective G2/M checkpoint and enhances radiosensitivity [27]. Generally, transcription activities of the E2F target are strictly regulated in the whole cell cycle [28]. However, abnormal expressions of E2F target genes are associated with cancer progression [28].

Currently, systemic chemotherapy is the main treatment approach for advanced NSCLC; however, its effectiveness has plateaued [29]. Immune checkpoint inhibitors (ICI) have been shown to increase the survival rates for advanced NSCLC patients [30]. Cancer cell growth and metastasis depend on the features of tumor cells and on interactions with the immune system [31]. Some ICI drugs, for example, ipilimumab, nivolumab, and pembrolizumab, have achieved remarkable results in clinical trials [32]. Our TIDE result showed that immunotherapeutic nonresponders have elevated NEIL3 expressions, indicating that targeting NEIL3 might improve the response rates of NSCLC patients to immunotherapy. Cisplatin and paclitaxel are core components of the NSCLC chemotherapy regimen. GDSC analysis showed that NEIL3 can significantly increase sensitivity to cisplatin and paclitaxel. Therefore, for patients with elevated NEIL3 levels, cisplatin- and paclitaxel-based chemotherapy might be more appropriate.

In summary, NEIL3 is overexpressed in NSCLC tissues and cell lines. In addition, patients with elevated NEIL3 expressions tended to have more aggressive clinical features and worse prognostic outcomes. *In vitro*, NEIL3 significantly facilitated NSCLC cell proliferation, invasion, and migration. GSEA showed that the pathway of PI3K/AKT/mTOR, G2/M checkpoints, and E2F target were abnormally activated in NEIL3 patients while western blot revealed that NEIL3 could partially activate the PI3K/AKT/mTOR signaling pathway, which might be responsible for the cancer-promoting effects of NEIL3. Finally, we found that NEIL3 could affect the sensitivity of NSCLC patients to immunotherapy and chemotherapy, making it a potential therapeutic target. Meanwhile, this study has some limitations. First, the patients obtained from TCGA were mainly white. Therefore, considering the potential racial bias, the applicability of our conclusions to other ethnicities would be unstable. Second, the clinical information for some patients was incomplete; for example, TNM classification of some patients was unknown. If all patients have complete clinical data, it would reduce the bias of our conclusions.

## Data Availability

Raw data is available from the corresponding author on reasonable request.
